# Supplementary material for: Q586B2 is a crucial virulence factor during the early stages of Trypanosoma brucei infection that is conserved amongst trypanosomatids
Source: Nat Commun. 2024 Feb 27;15:1779. doi: 10.1038/s41467-024-46067-4 (PMC10899635; doi:10.1038/s41467-024-46067-4)
Supplement: Supplementary file 1 — Supplementary Information [file 41467_2024_46067_MOESM1_ESM.pdf]

Supplementary information

Table S1: Overview of the different kinetoplast sequences used for the alignment and phylogenetic tree.

Species	Strain	Gene ID (TRiTRypDB or NCBI)	Uniprot protein ID
Trypanosoma brucei equiperdum	IVM-t1	DPX39_060045600	= same as Q586B2
Trypanosoma brucei equiperdum	IVM-t1	DPX39_020009200	= same as Q587H4
Trypanosoma brucei equiperdum	IVM-t1	DPX39_100119500	= same as Q389K9
Trypanosoma vivax	Y486	TVY486_0603600	G0TX79
Trypanosoma vivax	Y486	TVY486_0029080	/
Trypanosoma vivax	Y486	TVY486_1010980	G0U846
Trypanosoma congolense	IL3000	TcIL3000.A.H_000476700	F9WFX2
Trypanosoma congolense	IL3000	TcIL3000.A.H_000294200	/
Trypanosoma congolense	IL3000	TcIL3000.A.H_000818500	/
Trypanosoma evansi	STIB 805	TevSTIB805.6.4270	/
Trypanosoma evansi	STIB 805	TevSTIB805.2.1530	/
Trypanosoma evansi	STIB 805	TevSTIB805.10.11880	/
Trypanosoma brucei brucei	TREU 927	Tb927.6.4140	Q586B2
Trypanosoma brucei brucei	TREU 927	Tb927.2.2770	Q587H4
Trypanosoma brucei brucei	TREU 927	Tb10.26.0680	Q389K9
Trypanosoma brucei gambiense	DAL 972	Tbg972.6.3930	C9ZR85
Trypanosoma brucei gambiense	DAL 972	TbgDal_II1250	C9ZJ38
Trypanosoma brucei gambiense	DAL 972	Tbg972.10.13640	D0A4S3
Trypanosoma cruzi	CL Brener Non-Esmeraldo-like haplotype	TcCLB.511523.20	Q4D4V4
Trypanosoma cruzi	CL Brener Non-Esmeraldo-like haplotype	TcCLB.508739.90	Q4DNH1
Trypanosoma cruzi	CL Brener Non-Esmeraldo-like haplotype	TcCLB.424123.20	Q4CP97
Trypanosoma cruzi	CL Brener Non-Esmeraldo-like haplotype	TcCLB.507787.80	Q4DHS3
Trypanosoma cruzi	CL Brener Esmeraldo-like haplotype	TcCLB.511733.90	Q4D6Q6
Trypanosoma cruzi	CL Brener Esmeraldo-like haplotype	TcCLB.408437.20	Q4CPU3
Trypanosoma cruzi	CL Brener Esmeraldo-like haplotype	TcCLB.507711.20	Q4DZS6
Trypanosoma cruzi	CL Brener Esmeraldo-like haplotype	TcCLB.507625.120	Q4DPE4
Trypanosoma rangeli	AM80	TraAM80_07950	A0A3R7K0R9
Trypanosoma rangeli	AM80	TraAM80_05250	A0A422NFX4

Trypanosoma rangeli	AM80	TraAM80_06874	A0A422N867
Trypanosoma rangeli	AM80	TraAM80_03424	A0A422NPM1
Trypanosoma conorhini	025E	Tco025E_02289	A0A422Q5A3
Trypanosoma conorhini	025E	Tco025E_06843	A0A422NXI4
Trypanosoma conorhini	025E	Tco025E_09946	A0A3R7R3Z3
Trypanosoma conorhini	025E	Tco025E_03156	A0A422PWU3
Trypanosoma theileri	Edinburgh TM35	TM35_000391420	A0A1X0NKC2
Trypanosoma theileri	Edinburgh TM35	TM35_000072070	A0A1X0P259
Trypanosoma theileri	Edinburgh TM35	TM35_000035150	A0A1X0P760
Trypanosoma theileri	Edinburgh TM35	TM35_000083150	XP028884583
Leishmania donovani	LV9	LdBPK.30.2.002870	= same as A4I5W4
Leishmania donovani	LV9	LdBPK.02.2.000520	= same as A4HRQ0
Leishmania donovani	LV9	LdBPK.33.2.000660	= same as A4I8S7
Leishmania donovani	LV9	LdBPK.18.2.001640	= same as A4HY35
Leishmania major	SD 75.1	LMJSD75_300035600	Q4Q707
Leishmania major	SD 75.1	LMJSD75_020010800	E9ACC9
Leishmania major	SD 75.1	LMJSD75_330012700	Q4Q4E4
Leishmania major	SD 75.1	LMJSD75_180022500	Q4QDM4
Leishmania mexicanum	MHOM/GT/2001/U 1103	LmxM.29.2850	/
Leishmania mexicanum	MHOM/GT/2001/U 1103	LmxM.02.0550	/
Leishmania mexicanum	MHOM/GT/2001/U 1103	LmxM.32.0610	/
Leishmania mexicanum	MHOM/GT/2001/U 1103	LmxM.18.1640	/
Leishmania infantum	JPCM5	LINF_330013000	A4I8S7
Leishmania infantum	JPCM5	LINF_180022100	A4HY35
Leishmania infantum	JPCM5	LINF_300033900	A4I5W4
Leishmania infantum	JPCM5	LINF_020010800	A4HRQ0
Leishmania braziliensis	MHOM/BR/75/M29 04	LbrM.33.0650	A4HLA1
Leishmania braziliensis	MHOM/BR/75/M29 04	LbrM.30.2830	A4HIL9
Leishmania braziliensis	MHOM/BR/75/M29 04	LbrM.02.0520	A4H3E5
Leishmania braziliensis	MHOM/BR/75/M29 04	LbrM.18.1680	A4H9R6
Angomonas deanei	ATCC 30255	AGDE_02973	S9VEI0
Angomonas deanei	ATCC 30255	AGDE_00111	S9X5J9
Angomonas deanei	ATCC 30255	AGDE_04316	S9UIV2
Angomonas deanei	ATCC 30255	AGDE_14561	S9TQN6
Leptomonas pyrrhocoris	Isolate H10	LpyrH10_04_5660	A0A0M9G685
Leptomonas pyrrhocoris	Isolate H10	LpyrH10_27_0910	A0A0N0VD90
Leptomonas pyrrhocoris	Isolate H10	LpyrH10_13_2260	A0A0N0DUK3
Leptomonas pyrrhocoris	Isolate H10	LpyrH10_19_1230	A0A0N0VDW8
Leptomonas seymouri	ATCC 30220	Lsey_0209_0140	/
Leptomonas seymouri	ATCC 30220	Lsey_0531_0020	/
Leptomonas seymouri	ATCC 30220	Lsey_0320_0040	/
Leptomonas seymouri	ATCC 30220	Lsey_0304_0120	/
***Phytomonas* sp.**	Isolate EM1	GSEM1_T00006401001	W6KW07
***Phytomonas* sp.**	Isolate EM1	GSEM1_T00002717001	W6KDX4
***Phytomonas* sp.**	Isolate EM1	GSEM1_T00001357001	W6KQ71
***Phytomonas* sp.**	Isolate EM1	GSEM1_T00005005001	W6KVH2
Phytomonas serpens	9T	AIHY01003419	/
Phytomonas serpens	9T	AIHY01001458	/
Phytomonas serpens	9T	AIHY01002059	/
Phytomonas serpens	9T	AIHY01001404	/

Phytomonas francai	TCC 064	MJCC01000007	/
Phytomonas francai	TCC 064	MJCC01000003.1	/
Phytomonas francai	TCC 064	MJCC01000003.2	/
Phytomonas francai	TCC 064	MJCC01000006	/
Strigomonas culicis	ATCC 30268	STCU_00342	S9VLV9
Strigomonas culicis	ATCC 30268	STCU_00831	S9TXP6
Strigomonas culicis	ATCC 30268	STCU_00939	S9UQB1
Strigomonas culicis	ATCC 30268	STCU_00096	S9WDJ7
Strigomonas galati	TCC219	AUXN01000505	/
Strigomonas galati	TCC219	AUXN01000135	/
Strigomonas galati	TCC219	AUXN01003365	/
Strigomonas galati	TCC219	AUXN01002257	/
Strigomonas oncopelti	TCC290E	AUXK01000203	/
Strigomonas oncopelti	TCC290E	AUXK01002482	/
Strigomonas oncopelti	TCC290E	AUXK01000974	/
Strigomonas oncopelti	TCC290E	AUXK01005767	/
Herpetomonas muscarum	TCC001E	AUXJ01001970	/
Herpetomonas muscarum	TCC001E	AUXJ01001978	/
Herpetomonas muscarum	TCC001E	AUXJ01002588	/
Herpetomonas muscarum	TCC001E	AUXJ01001786	/
Lotmaria passim	ATCC PRA-422	MDUF01002963	/
Lotmaria passim	ATCC PRA-422	MDUF01004965	/
Lotmaria passim	ATCC PRA-422	MDUF01003315	/
Lotmaria passim	ATCC PRA-422	MDUF01001977	/
Crithidia mellificae	ATCC 30862	MDUD01002396	/
Crithidia mellificae	ATCC 30862	MDUD01008845	/
Crithidia mellificae	ATCC 30862	MDUD01002065	/
Crithidia mellificae	ATCC 30862	MDUD01005607	/
Crithidia bombi	VT1	MDUG01005950	/
Crithidia bombi	VT1	MDUG01005901	/
Crithidia bombi	VT1	MDUG01001467	/
Crithidia bombi	VT1	MDUG01001463	/
Crithidia fasciculata	Cf-Cl	CFAC1_260052900	/
Crithidia fasciculata	Cf-Cl	CFAC1_160016900	/
Crithidia fasciculata	Cf-Cl	CFAC1_140028700	/
Crithidia fasciculata	Cf-Cl	CFAC1_280015200	/
Endotrypanum monterogeii	LV88	EMOLV88_300034000	/
Endotrypanum monterogeii	LV88	EMOLV88_020008100	/
Endotrypanum monterogeii	LV88	EMOLV88_180020600	/
Endotrypanum monterogeii	LV88	EMOLV88_330010900	/
Paratrypanosoma confusum	CUL13MS	PCON_0025060	/
Paratrypanosoma confusum	CUL13MS	PCON_0060150	/
Paratrypanosoma confusum	CUL13MS	PCON_0045870	/
Paratrypanosoma confusum	CUL13MS	PCON_0070360	/
Bodo saltans	"Lake Konstanz"	BSAL_18095	A0A0S4JFI1
Bodo saltans	"Lake Konstanz"	BSAL_62145	A0A0S4IRP9
Bodo saltans	"Lake Konstanz"	BSAL_66355	A0A0S4IPF2
Bodo saltans	"Lake Konstanz"	BSAL_09835	A0A0S4JBB4

Footnotes:

a) Homologues of *Tb927.6.4140* were identified using tBLASTn searches to screen a wide range of eukaryote genomes in the NCBI and TriTrypDB databases.

b) If no data is available it is shown as “/”.

Table S2: Overview of the PCR long primers used for the *in-situ* tagging.

Gene	Forward	Reverse
***Tb*927.9.12550 (Glycerol kinase)**	5′-CCGACCTGTAGGAGAGTTAGCCACTAAGTTTATTATTATCATTGTTTCAAGAAAGACAAAACTATCCGTAAACGAGAAAAGTATAATGCAGACCTGCTGC-3′	5′-GAGACGGGGCGCTGCCGCTCATCGAAGATGATGAAGCGGGTGCTGGTTGTTCCCTGGTCAATGGATCCGACGTACTTCATACTACCCGATCCTGATCC-3′
***Tb*927.3.800 (VIT1)**	5′-TTTTCTTGTAACAACGTGAAATAGGGAAATAAAGCCCCTTGCATTAATTTTGGTCCAGTGAAACATCTTCTTGACTGACAGTATAATGCAGACCTGCTGC-3′	5′-GTTTCAAAGGCCTTTCGGGCGGCCGATATGCTCTTATAATTCCTTGCCCCACTTACATCAATTTTTTCTGATTCGGACATACTACCCGATCCTGATCC-3′
***Tb*927.11.15240 (Rab2B)**	5′-GTTCTTTTTTTTCTTTTTTTTATTATTGAGGAGAACAGTAACAACAACAACAGCAACAACAAAACGTCCAGACACCGCAGTATAATGCAGACCTGCTGC-3′	5′-GTGAACTGCAGTAGGAGGCAACTCTTTCCCACTCCGCTATCACCAATAATAATGTACTTGAAAACATAATGGTGCTGCTGACTACCCGATCCTGATCC-3′
***Tb*927.11.4570 (Rab5B)**	5′-AGCTAGAGTGGACAAACGTCCCCCCCTTTTTGGCGTGCATCAATCTATTACATAGTGAGCTGGATAACTTTCTCTGTCCGTATAATGCAGACCTGCTGC-3′	5′-AGGGATGATTTACCAACACCGCTATCGCCCAAAAGGACAATTTTGTACTTCTTTGTTGGGGCGGCAAGCGTCTTCACAGAACTACCCGATCCTGATCC-3′
***Tb*927.8.4330 (Rab11)**	5′-TATTTGAAGGGAAGGAAGAGGAGAGTTCTCGCAGAACCCATATCACGGTTTTAATATCTTTCATACTCAGTAAAACCAGAAACAGTATAATGCAGACCTGCTGC-3′	5′-GTGTAGCGAGTCATGAGGTTGGACTTCCCAACACCGCTGTCACCCACGATAACAACTTTGAACGTAAGGTTCATGTCTTCACTACCCGATCCTGATCC-3′

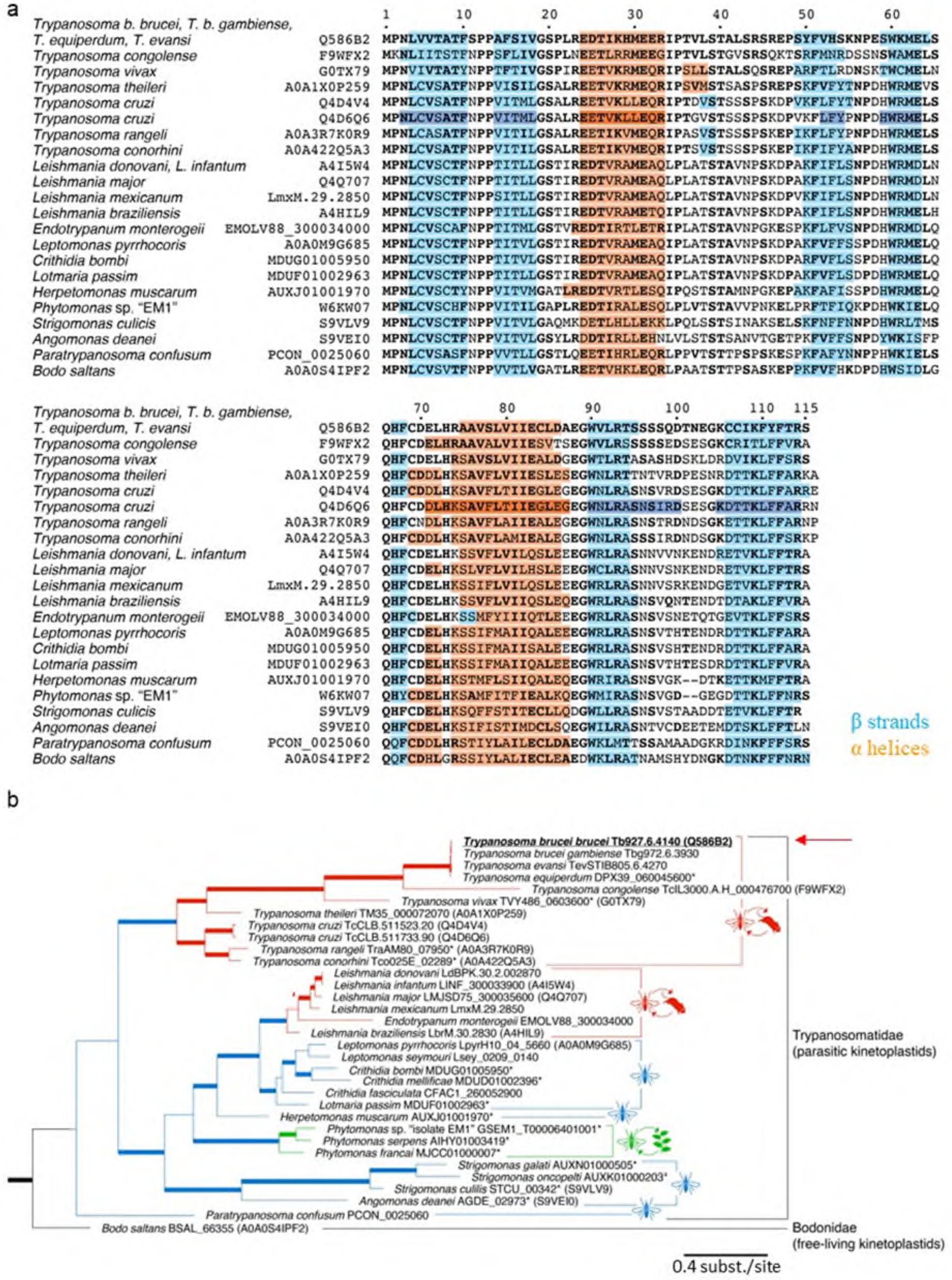

Fig. S1: The *Tb927.6.4140* gene and Q586B2 protein are evolutionary conserved within the related families Trypanosomatidae and Bodonidae. a) Comparative alignment of Q586B2 with Q4D6Q6 and other proteins as encoded by closest related homologous genes

found in other kinetoplastid taxa. Sequence similarity to Q586B2 is visualised by typing identical aligned amino acids in boldface. Note that some sequences occur in multiple species (i.e. these species share identical proteins). Secondary structures predicted by Phyre^2 include both beta strands (light blue segments) and alpha helices (light orange segments), revealing the evolutionary conservation of this structure. The known secondary structure of Q4D6Q6 is shown by darker shades of both colors. Phyre^2 used this protein as prediction template for all other proteins. **b**) Evolutionary gene tree for *Tb927.6.*4140 of *Trypanosoma brucei brucei* and orthologous sequences found in other Trypanosomatidae and Bodonidae. The pictured tree corresponds to one of four gene clades reconstructed by Bayesian phylogeny inference based on 126 homologous gene sequences (See Fig. S2 for the full tree). Branches supported by posterior probabilities >0.95 are drawn as thick lines. Evolution of life histories across the tree inferred from present-day life histories is represented by branch color variation and corresponding icons: blue branches indicate a parasitic (monoxenous) life history involving an arthropod (insect) host; green and red branches indicate dixenous life histories, with respectively plants and mammals as secondary hosts. Genes are labelled by their accession number in TRiTRypDB (numbers without asterisk) or NCBI Genbank (numbers with asterisk). Labels between brackets represent corresponding Uniprot accession numbers when available.

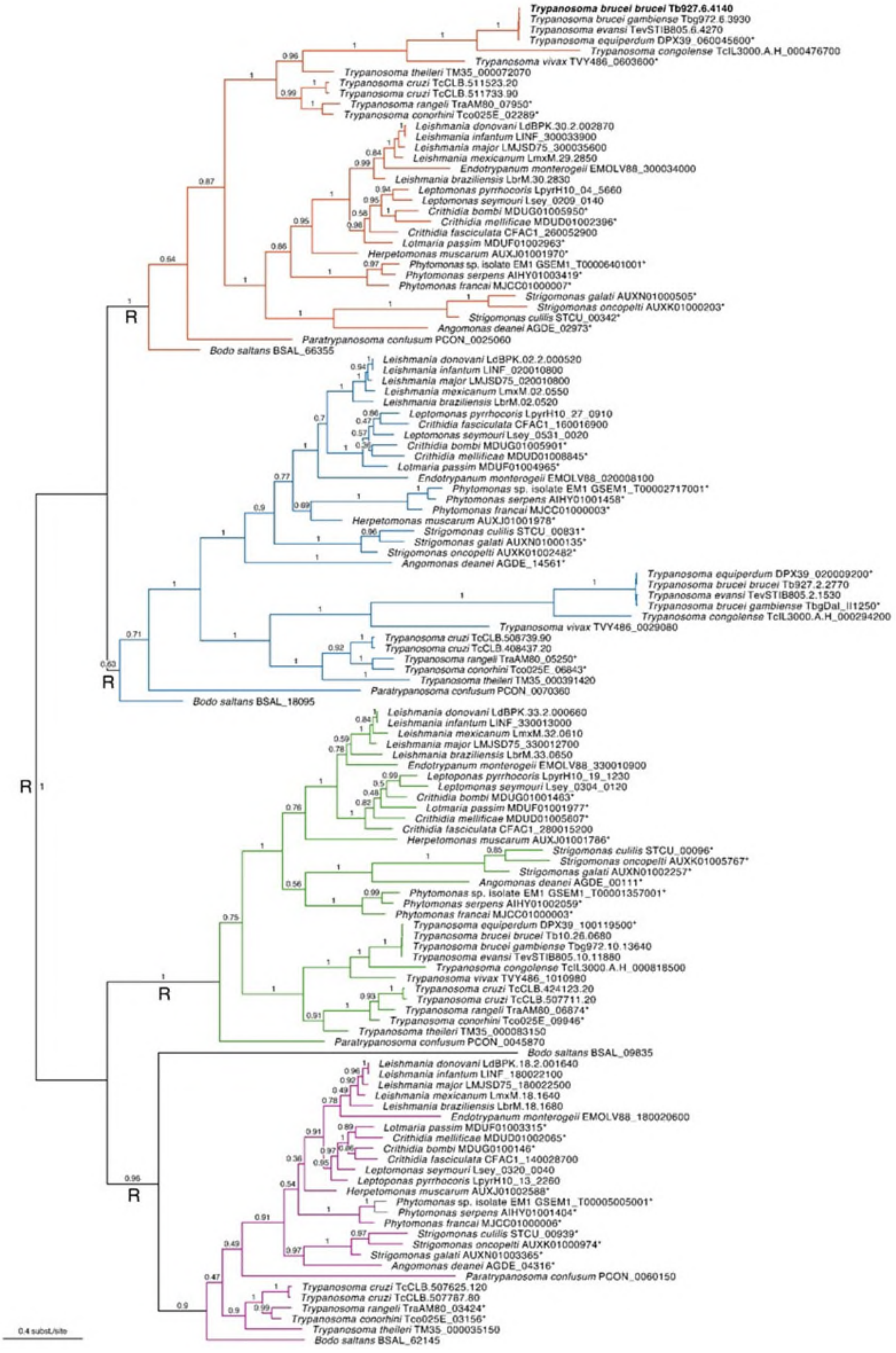

Fig. S2: Phylogenetic tree for *Tb927.6.4140* of *Trypanosoma b. brucei* and 125 homologous gene sequences found in a wide range of Trypanosomatidae and Bodonidae. The depicted tree represents the Bayesian consensus phylogram; numbers on branches represent Bayesian posterior probabilities. In the absence of reliable outgroup sequences (homologous sequences

for which a basal divergence with these 126 sequences is certain), the tree is *a priori* unrooted. However, a root position on any of the branches indicated with ‘R’ would implicate the least number of gene duplication events and is therefore the most parsimonious evolutionary scenario. Genes are labelled by their accession number in TRiTRypDB (numbers without asterisk) or NCBI Genbank (numbers with asterisk) and shown in Table S1.

a

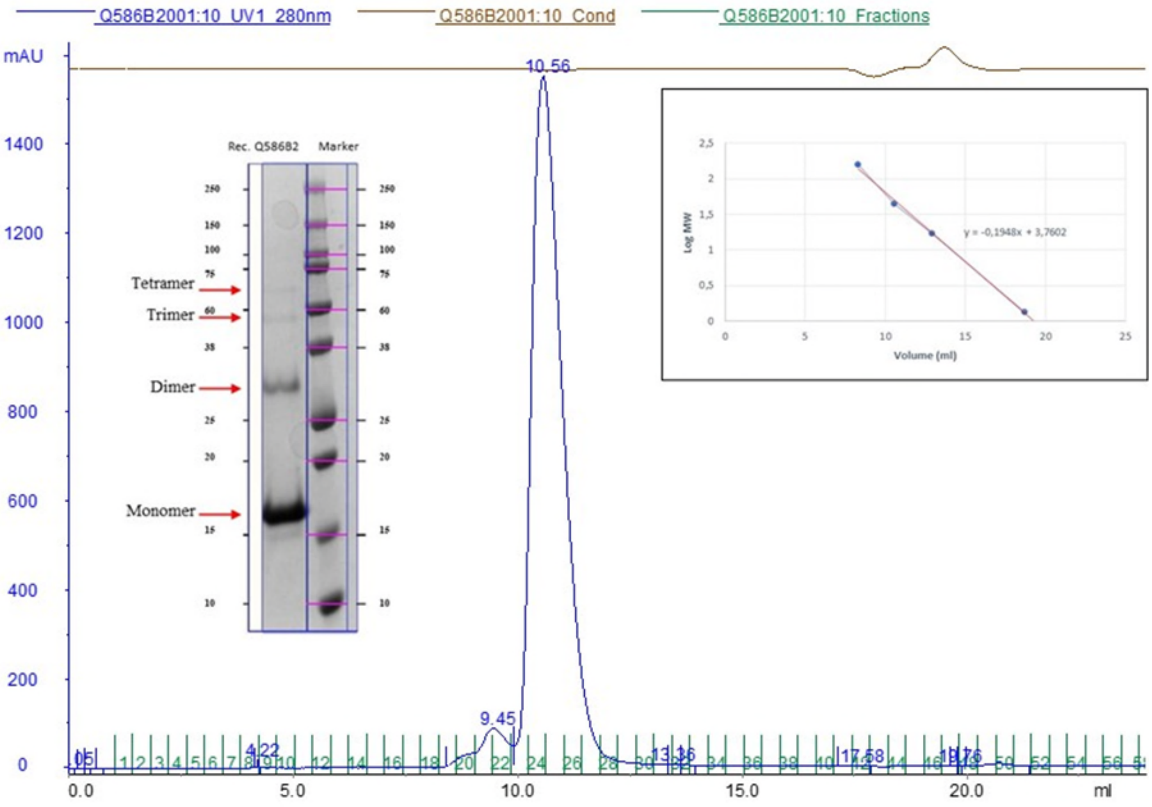
Q586B2001:10_UV1_280nm
Q586B2001:10_Cond
Q586B2001:10_Fractions
mAU
1400
1200
1000
800
600
400
200
0
10.56
9.45
0.0
5.0
10.0
15.0
20.0
ml
Rec. Q586B2
Marker
Tetramer
Trimer
Dimer
Monomer
Log MW
Volume (ml)
y = -0,1948x + 3,7602

b

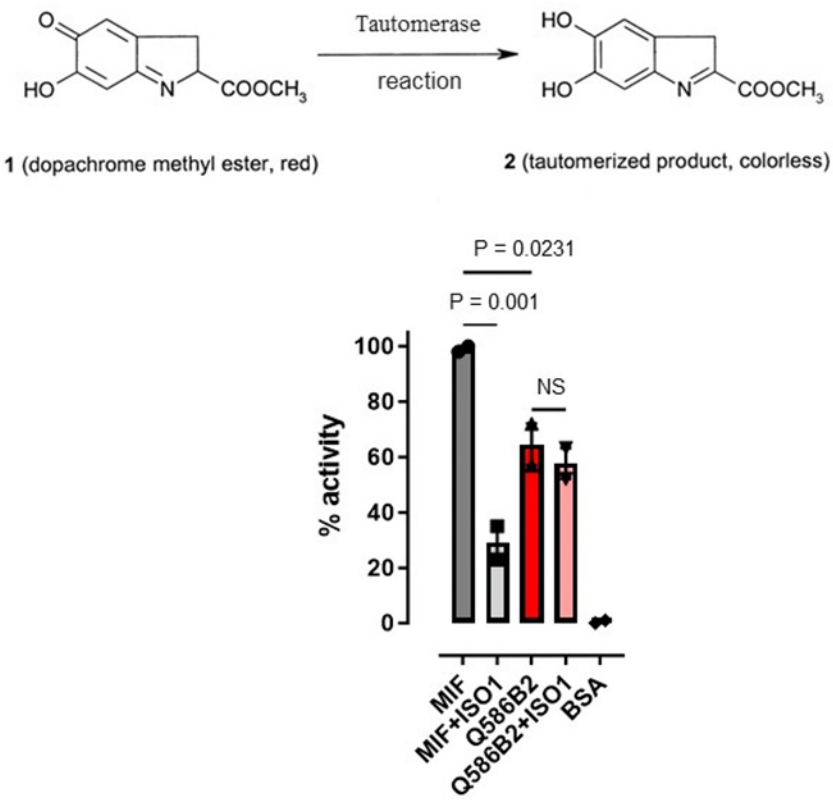
Tautomerase
reaction
1 (dopachrome methyl ester, red)
2 (tautomerized product, colorless)
P = 0.0231
P = 0.001
NS
% activity
MIF
MIF+ISO1
Q586B2
Q586B2+ISO1
BSA

Fig. S3: Functional characterization of the Q586B2 protein. a) Purity of recombinant Q586B2. **a)** FPLC chromatogram of the purified Q586B2 protein run on a superdex75 (10/300) column equilibrated against PBS (Äkta explorer10s/GE Healthcare). To determine the molecular weight of Q586B2 a standard (BiaRad) was run onto the column and the elution volume of each standard was plotted against the log10 of the molecular weight of the standards. A linear regression allows determination of the molecular weight of Q586B2 (~55 kDa) which corresponds to the size of a tetrameric protein. A 12% SDS-PAGE of the purified Q586B2 protein under reducing conditions reveals that the majority of the protein consists predominantly as a monomer of about 17 kDa and to a lesser extend as a multimer (dimer, trimer and tetramer) (indicated by red arrows). **b**) Q586B2 harbors tautomerase activity. The tautomerase reaction converts the red dopachrome methyl ester (measured at OD_{475nm}) into its colorless tautomerized product. Hence, the tautomerase activity was determined by the semi-continuous reduction in signal at OD_{450nm}, in the presence of recombinant mouse MIF (rmMIF), rmMIF + its inhibitor ISO-1 (positive controls), Q586B2, Q586B2 + ISO-1 and BSA (negative control). Graphs represent the percentage tautomerase activity compared to that of rmMIF alone (set at 100% activity). Data are shown as mean ± SEM and are representative of 3 independent experiments (n = 2). Data are analyzed via one way ANOVA using Turkey's multiple comparison test with ns indicating not significant for $p \geq 0.05$ and $p < 0.05$. Source data are provided as a Source Datafile.

a (i)

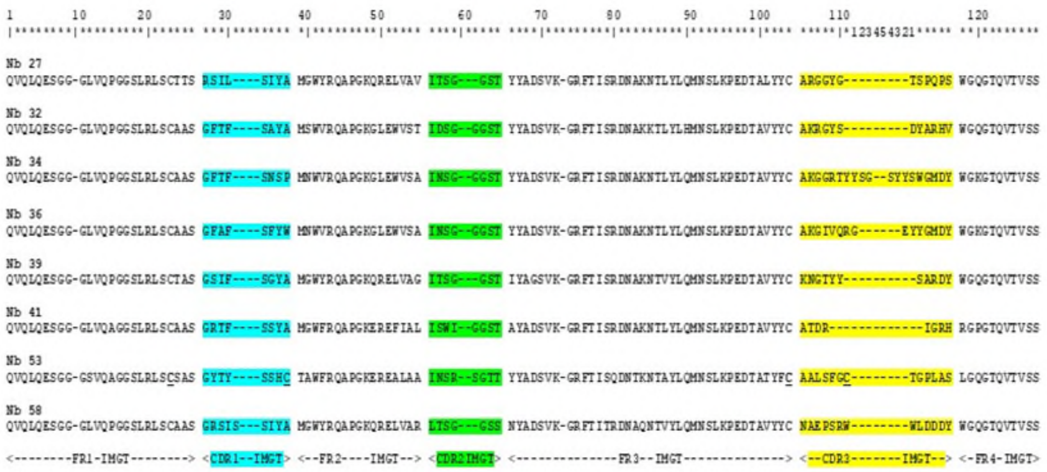

(ii)

Clone Name	Nr. aa	Molecular Weight (Da)	pI	ε (M-1 cm-1)	Kd (nM)
Nb 27	137	14901,51	8,91	27390	52
Nb 32	138	15050,62	8,55	32890	46,6
Nb 34	145	15806,37	8,82	41370	36,7
Nb 36	141	15512,2	7,95	39880	11,3
Nb 39	136	14732,24	8,88	28880	49,9
Nb 41	134	14607,21	8,98	22920	14,5
Nb 53	139	14802,17	7,19	18910	45,5
Nb 58	139	15341,8	6,64	36900	36,7

b

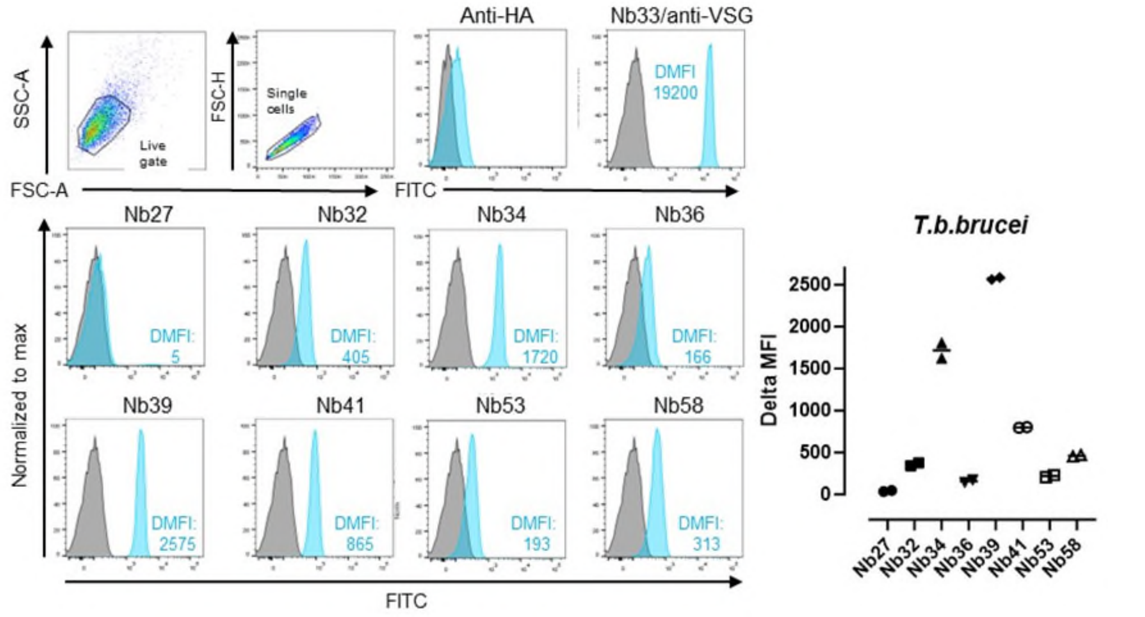

Fig. S4: Characteristics of Q586B2 specific Nbs and determination of their binding potential on fixed and permeabilized *T. brucei* parasites. a) (i) Sequence alignment of the 8 different anti-Q586B2 Nbs, which are organized into different families according to Kabat classification.[49] Complementarity determinant region 1 (CDR1), CDR2, and CDR3 are indicated in blue, green, and yellow, respectively. (ii) Characteristics of the 8 different anti-

Q586B2 Nbs as well as the binding affinities towards Q586B2 in ELISA. **b**) (left panels) Representative flow cytometric profile of purified, fixed and permeabilized *T. b. brucei* (Antat1.1E) parasites plotted in an SSC-A versus FSC-A plot and subsequently gating on single cells (FSC-H versus FSC-A plot). 10^6 parasites in presence or absence of 5 µg of HA-tagged Nb was used per condition. Detection of the Nbs was achieved using an Alexa-488 labelled anti-HA IgG (1 µg). Signals of parasites in presence of these Alexa-488 labelled anti-HA IgG alone was used as negative control, while Nb33 (anti-VSG Nb) was used as a positive control. (right panel) Bar chart showing the median fluorescence intensity (MFI) of the different anti-Q586B2 Nbs following binding to fixed and permeabilized *T. b. brucei* AnTat1.1E parasites. Each condition consists of technical duplicates (n = 2) and results are shown ± median. Source data are provided as a Source Datafile.

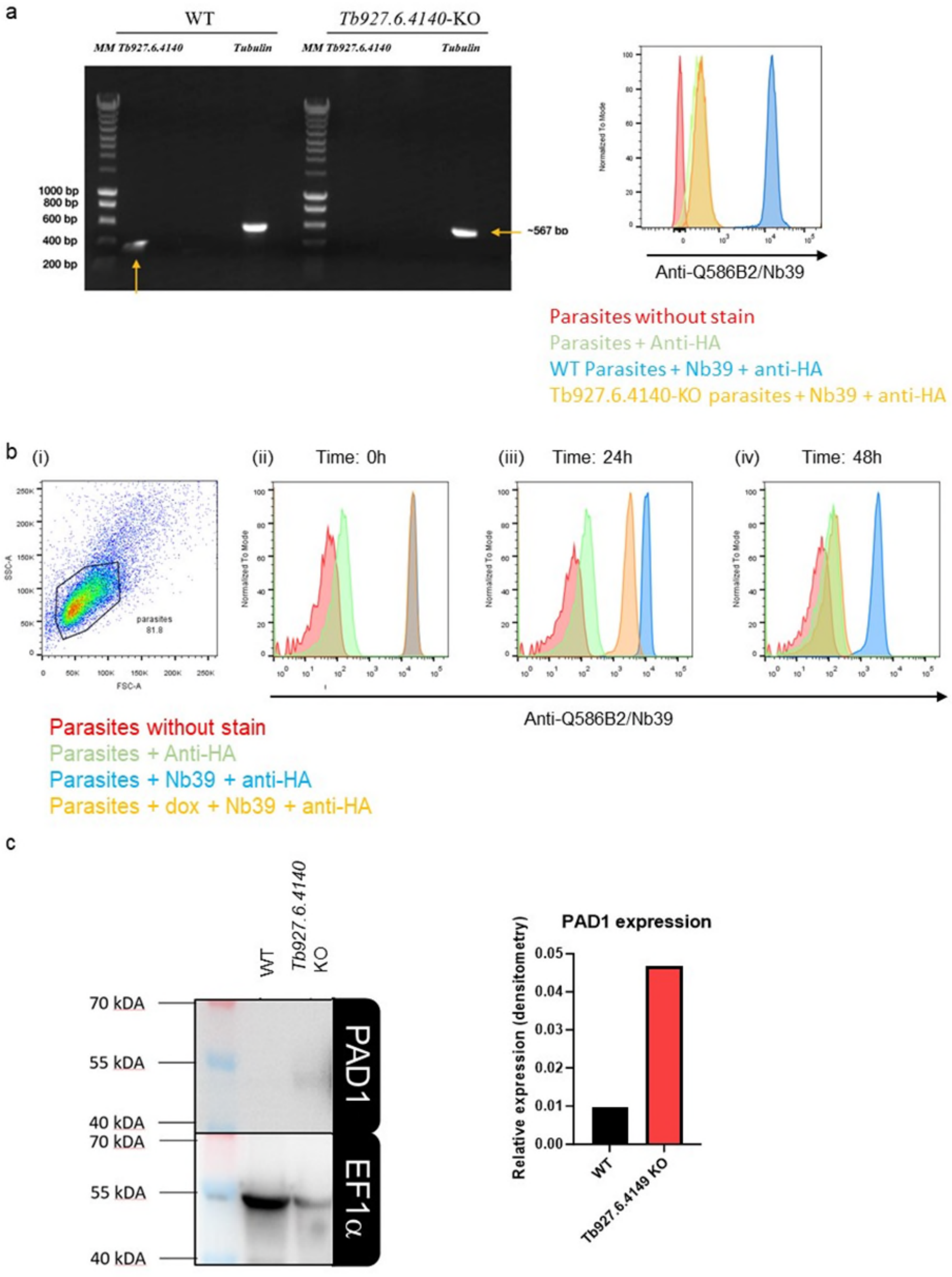

Fig. S5: Generation of full *Tb927.6.4140*-deficient parasites as well as determination of the kinetics of *Tb927.6.4140* knock-down upon doxycycline treatment *in vitro* and PAD1 expression on purified parasites. a) (left panel) Representative PCR results confirming the

efficient generation of full *Tb927.6.4140*-deficient parasites. WT (90:13) and *Tb927.6.4140*-KO bloodstream *T. b. brucei* parasites were grown *in vitro* in HMI-9 medium. PCR was performed on both parasite strains to detect the *Tb927.6.4140* gene. As control the *tubulin* gene was used. The specific primers for *Tb927.6.4140* and *tubulin* are shown in the STAR methods section. In WT parasites an amplicon of 348bp corresponding to the *Tb927.6.4140* gene could be detected, while in the *Tb927.6.4140*-KO parasites no amplicon was detected. Of note, the tubulin with expected size of 567 bp was detected in both WT and *Tb927.6.4140*-KO bloodstream *T. b. brucei* parasites. (right panel) Detection of Q586B2 expression in WT (90:13, blue) and *Tb927.6.4140-KO* parasites (orange) using the gating strategy in b (i). **b**) Detection of Q586B2 expression in WT and *Tb927.6.4140-KD* parasites upon exposure to doxycycline. i) Representative gating strategy used to detect trypanosomes via flow cytometry. (ii-iv) WT (90:13) and inducible *Tb927.6.4140-KD* parasites were kept for 0, 24 or 48 hours under doxycycline pressure (orange) or without doxycycline (blue). Subsequently, parasites were fixed and permeabilized and Q586B2 detection was established using Nb39 followed by anti-HA-Alexa488 staining. Controls used are no staining (red) and anti-HA staining alone (green). Results are representative of 3 independent experiments. **c**) Detection of PAD1 expression in purified *T. brucei brucei* (90:13) and *Tb927.6.4140*-KO parasites isolated at peak parasitemia via western blot. EF1α protein expressions was used to normalize the expression of PAD1. Next, the intensity of the bands was quantified via densitometry using a Vilber Fusion Imager. Source data are provided as a Source Datafile.

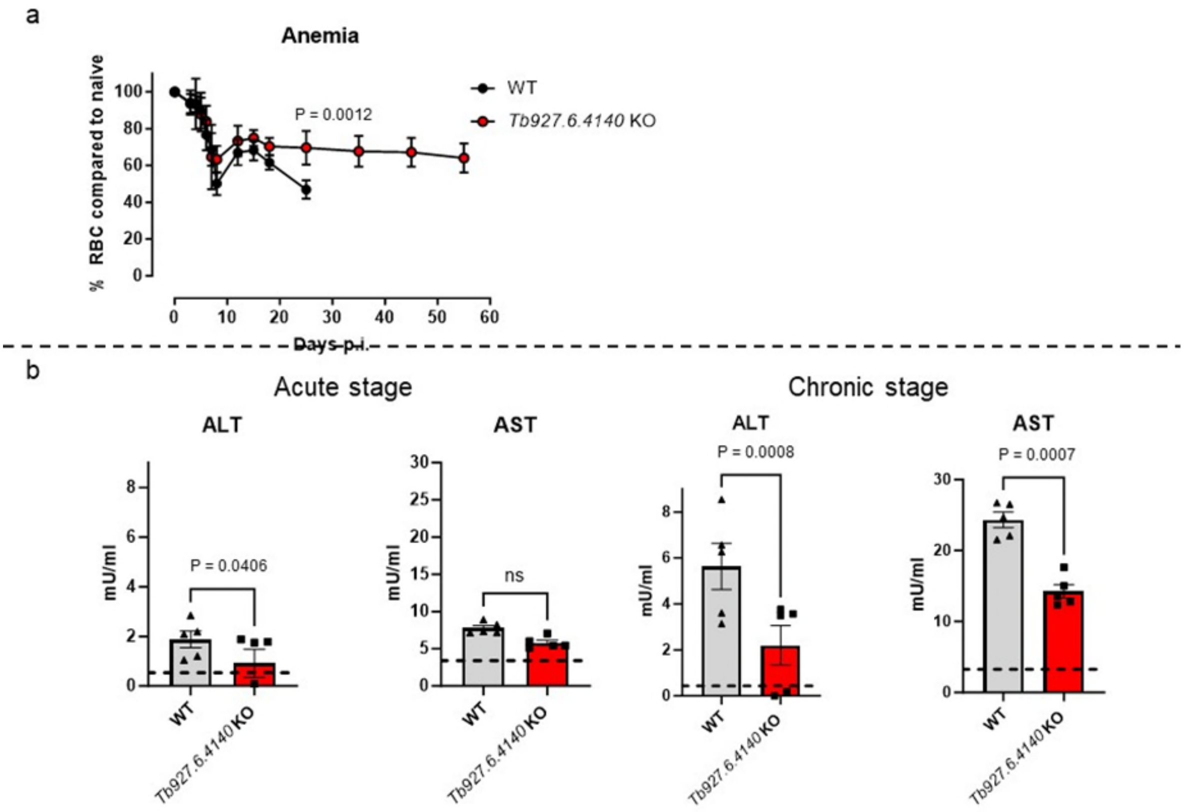

Fig. S6: *Tb927.6.4140* deficiency coincides with a reduced AT-associated immunopathology development during the acute and chronic stage of infection. Mice were injected intraperitoneally with 5000 WT (90:13 strain) parasites or 5000 *Tb927.6.4140*-KO parasites. a) Anemia development in wild type parasite (WT, black box) or *Tb927.6.4140*-KO parasite (red box) infected mice during the course of infection. (b) At 6 days post injection acute stage) and at 22 days post injection (Chronic stage) the serum ALT and AST levels of wild type parasite (WT, grey box) or *Tb927.6.4140*-KO parasite (red box) infected mice measured. Results are representative of 2 independent experiments (n = 5) and presented as mean ± SEM. Paired Student's t-tests were used for comparison between experimental groups, with ns indicating not significant for p≥0.05 and p<0.05. Source data are provided as a Source Datafile.

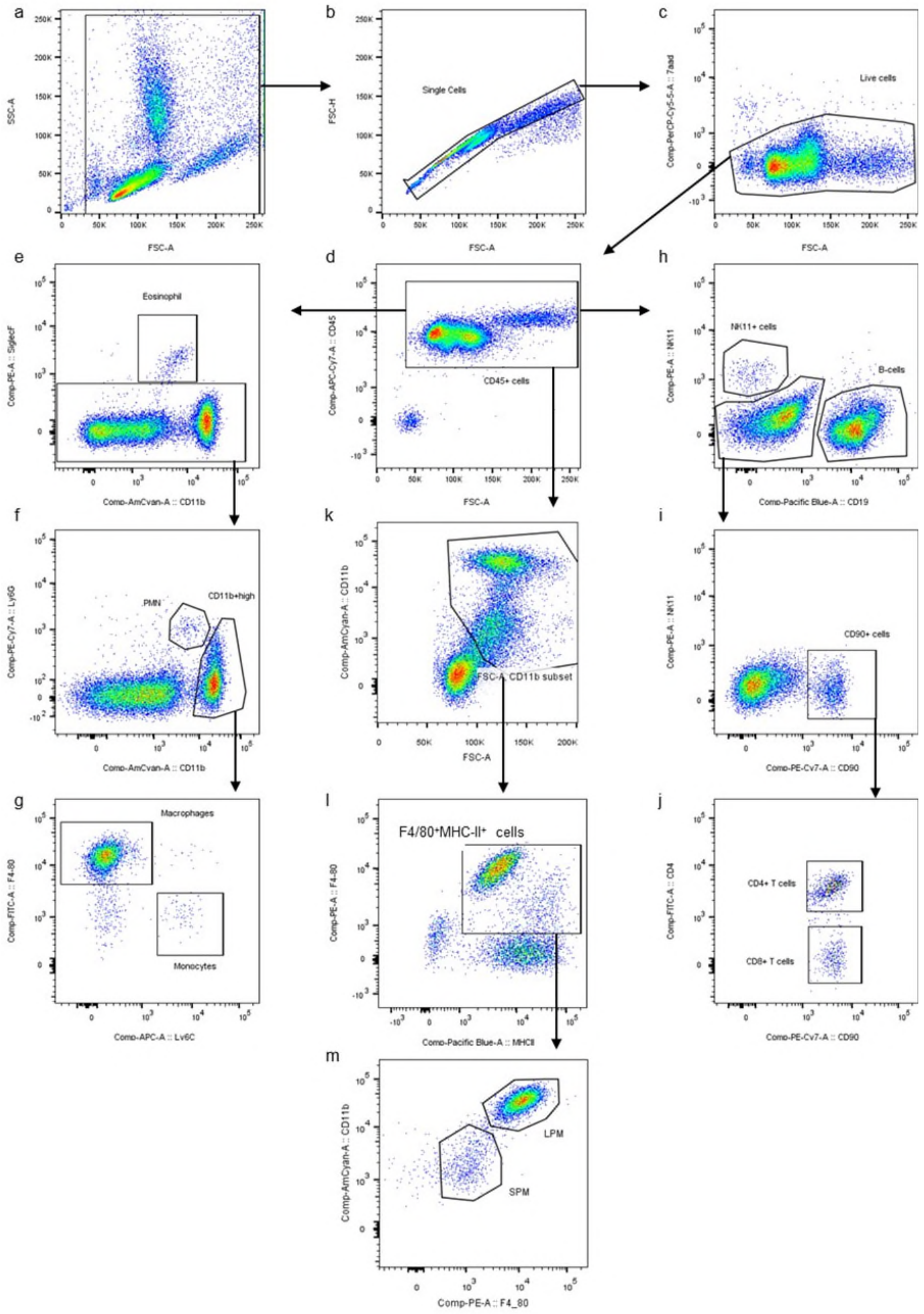

Fig. S7: Representative gating strategy used to identify different cell populations within the peritoneal lavage. Within the SSC-A versus FSC-A plot, a live gate (exclusion of debris)

is selected (**a**). Next, a SSC-A versus SSC-H plot allows the selection of single cells (**b**), followed by selection of living cells using an 7AA-D versus FSC-A plot (**c**). Within the live, single cells, $CD45^+$ cells represent all hematopoietic cells (**d**). Within the $CD45^+$ gate, a CD11b versus SiglecF plot allows identification of eosinophils ($CD11b^{int}$ $SiglecF^{hi}$ cells) represent eosinophils (**e**). Following exclusion of eosinophils (*i.e.* rest), a CD11b versus Ly6G plot allows identification of neutrophils (PMN, $CD11b^{int}$ $Ly6G^+$ cells) and $CD11b^{high}$ cells (**f**). Subsequently, a plot of F4/80 versus Ly6c allows identification of $CD11b^{hi}$ $F4/80^+$ cells represent macrophages and $CD11b^{hi}$ $Ly6C^{hi}$ $Ly6G^-$ cells represent monocytes (**g**). Within the $CD45^+$ gate, a CD19 versus NK11 plot allows identification of $NK1.1^+$ cells representing NK/NKT cells and $CD19^+$ cells represent B lymphocytes (**h**). Following exclusion of $NK1.1^+$ cells and $CD19^+$ cells (*i.e.* rest 2), $CD90^+$ cells (T-lymphocytes) were selected in a NK11 versus CD90 plot (**i**). Next, within the $CD90^+$ cells, $CD4^+$ $NK1.1^-$ $CD90^+$ T cells (referred by as CD4 T cells) and $CD4^-$ $NK1.1^-$ $CD90^+$ T cells (referred by as CD8 T cells) were identified (**j**). Within the $CD45^+$ gate, a CD11b versus FSC plot allows selecting $CD11b^+$ cells (**k**). These cells are put in an F4/80 versus MHC-II plot allowing selection of $F4/80^+MHC\text{-}II^+$ cells (**l**). The selected cells are put in a CD11b versus F4/80 plot to identify large peritoneal macrophages (LPM, $CD11b^{+high}$, $F4/80^{+high}$) and small peritoneal macrophages (SPM, $CD11b^{+int/low}$, $F4/80^{+int/low}$) (**m**).

a

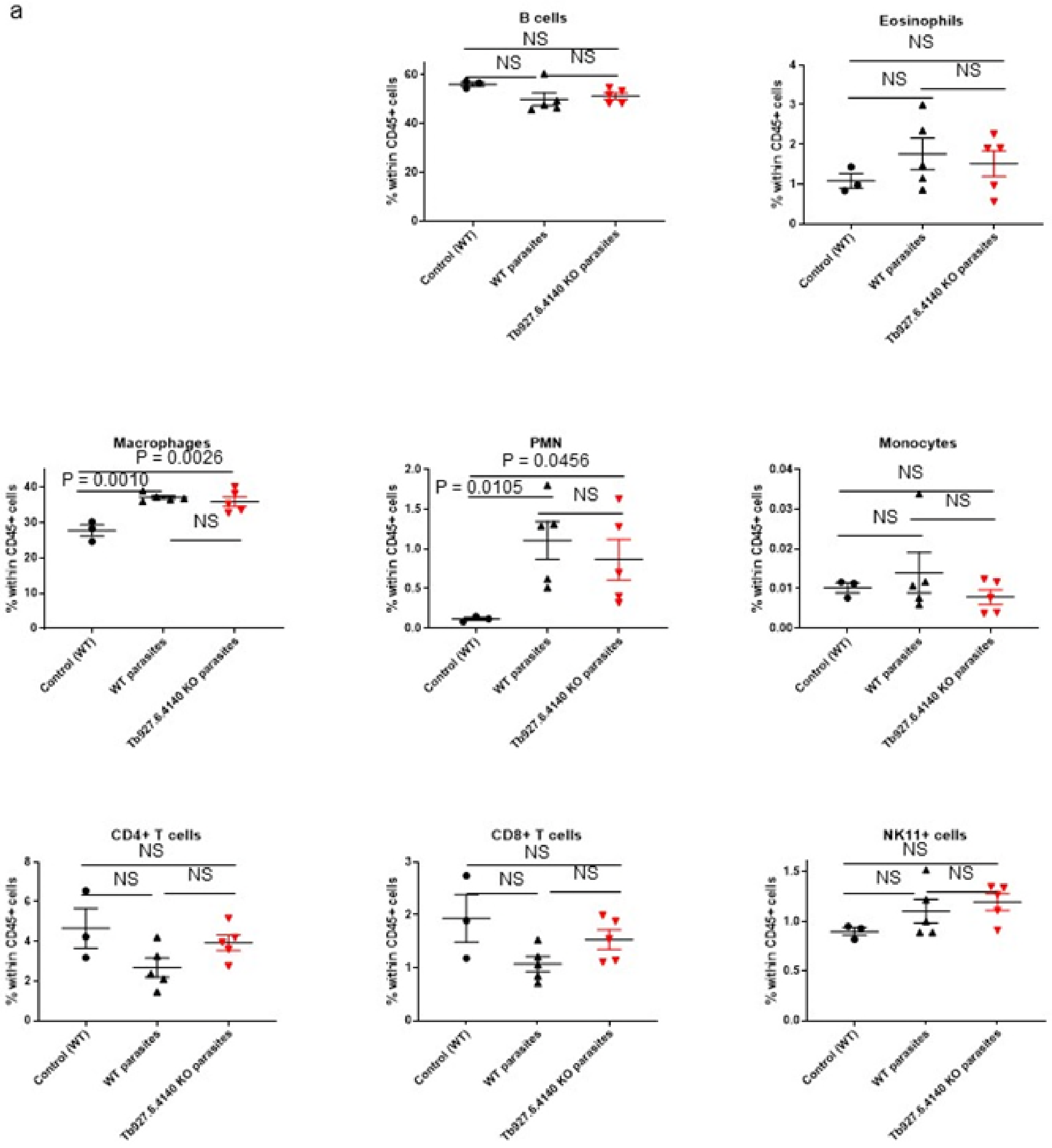
B cells
Eosinophils
Macrophages
PMN
Monocytes
CD4+ T cells
CD8+ T cells
NK11+ cells
% within CD45+ cells
Control (WT)
WT parasites
Tb927.6.4140 KO parasites
NS
P = 0.0026
P = 0.0010
P = 0.0456
P = 0.0105

b

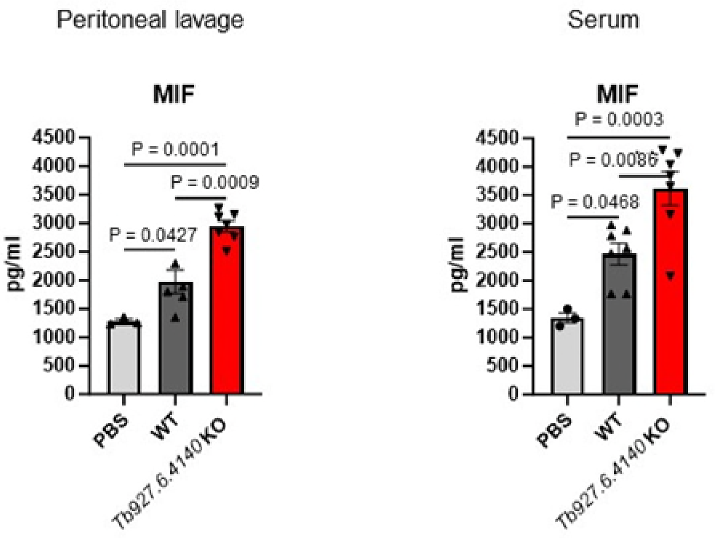
Peritoneal lavage
Serum
MIF
pg/ml
P = 0.0001
P = 0.0009
P = 0.0427
P = 0.0003
P = 0.0086
P = 0.0468
PBS
WT
Tb927.6.4140 KO

Fig. S8: Percentage of different immune cell subsets within the peritoneum as well as MIF cytokine levels in peritoneum and serum in naïve (control), WT and *Tb927.6.4140*-KO parasite infected mice. C57BL/6 mice were injected intraperitoneally with PBS (control), 5000 WT (90:13 strain) parasites or 5000 *Tb927.6.4140*-KO parasites and 18 hours later mice were sacrificed, and the peritoneal cells isolated. Of note, all injections consisted of 200 µl. **a**) The cellular composition of the peritoneal cells, *i.e.* B-cell, eosinophils, macrophages, monocytes, PMN (polymorphonuclear cells), $CD4^+$ and $CD8^+$ T cells, and NK cells, was determined using the gating strategy described in Fig. S6. **b**) The cytokine concentration in peritoneal lavage (left panel) and serum (right panel) were determined via an MSD kit and only for MIF detectable levels were recorded. Results are representative of 2 independent experiments (n = 3 for the control and n = 5 for the infected groups) and presented as mean ± SEM. A one-way ANOVA with Turkey's multiple comparison test was performed. NS: Not significant. Source data are provided as a Source Datafile.

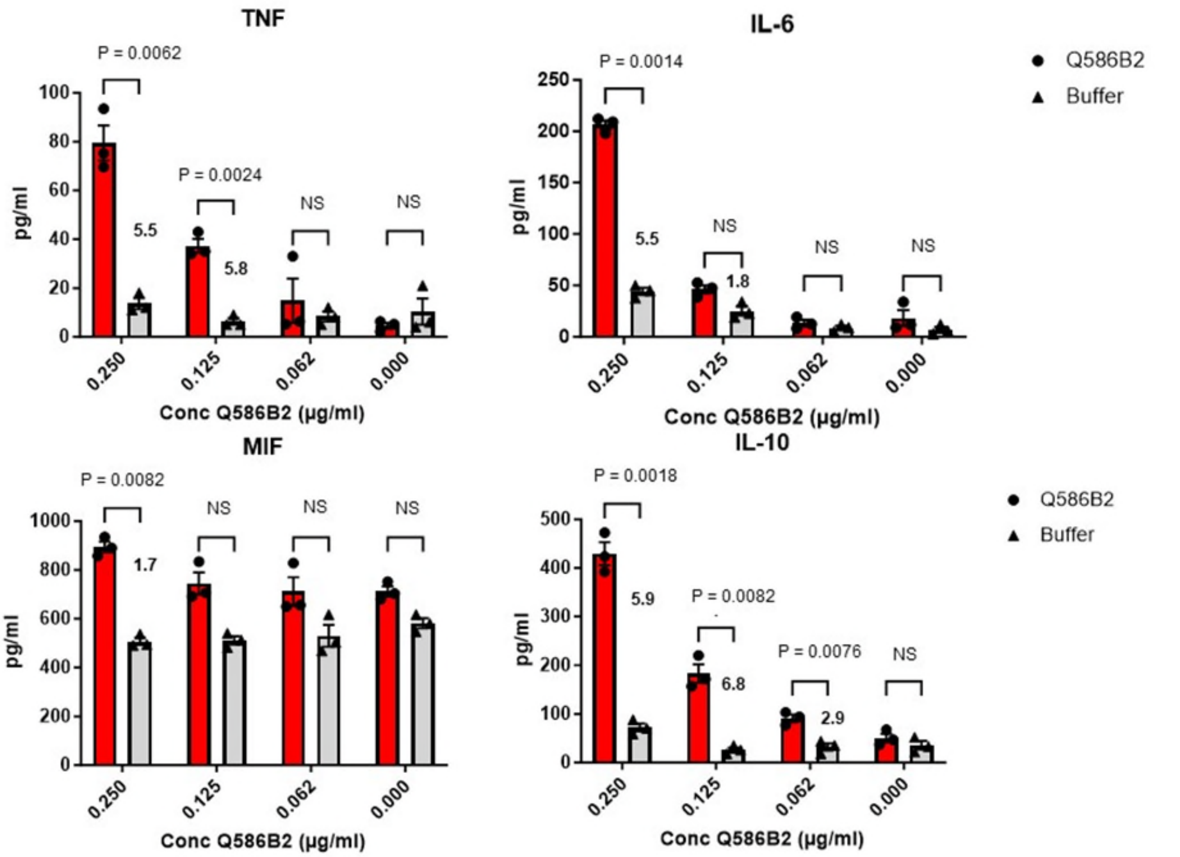

Fig. S9: Q586B2 exerts both pro- and anti-inflammatory activity on myeloid cells (PECs) *in vitro*. Production of TNF, IL-6, MIF and IL-10 by PECs isolated from C57BL/6 mice (2 * 10^5 cells/well) following 48 hours incubation with a ½ serial dilution of LPS-free Q586B2 (red bars) starting from 0.25 µg/ml or buffer alone (grey bars). Negative controls: cells incubated with buffer alone or without any protein. Data are shown as means ± SEM and representative of 2 independent experiments (n = 3). Data are analyzed using a multiple paired Student's t-test, with ns indicating not significant for $p \geq 0.05$ and $p < 0.05$. Source data are provided as a Source Datafile.

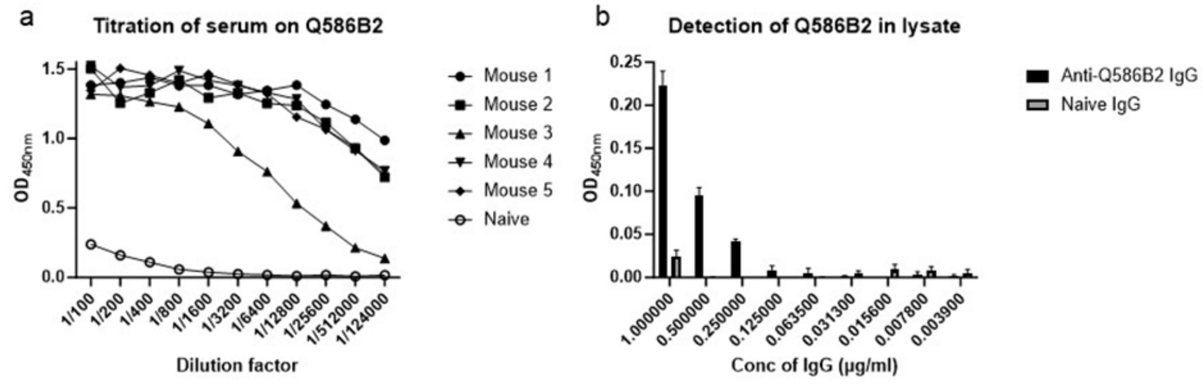

Fig. S10: Q586B2 immunization triggers a strong anti-Q586B2 humoral response whereby the IgGs are able to recognize the native antigen in *T. b. brucei* lysate. a) Antibody (IgG) response in serum from individual Q586B2-treated and naïve mice tested in ELISA on Q586B2 coated antigen. Naïve represent a pool of 5 mice that only received the mock treatment. **b**) Purified IgGs from Q586B2- and mock immunized mice are tested on lysate of *T. b. brucei* (AnTat1.1E) parasites in ELISA. Technical triplicates were used.
