# Supplementary material for: Q586B2 is a crucial virulence factor during the early stages of Trypanosoma brucei infection that is conserved amongst trypanosomatids
Source: Nat Commun. 2024 Feb 27;15:1779. doi: 10.1038/s41467-024-46067-4 (PMC10899635; doi:10.1038/s41467-024-46067-4)
Supplement: Supplementary file 3 — Reporting Summary [file 41467_2024_46067_MOESM3_ESM.pdf]

nature portfolio

Corresponding author(s): Prof. Dr. ir. Stijlemans Benoit

Last updated by author(s): 22-01-2024

Reporting Summary

Nature Portfolio wishes to improve the reproducibility of the work that we publish. This form provides structure for consistency and transparency in reporting. For further information on Nature Portfolio policies, see our Editorial Policies and the Editorial Policy Checklist.

Statistics

For all statistical analyses, confirm that the following items are present in the figure legend, table legend, main text, or Methods section.

n/a	Confirmed	
☐	☒	The exact sample size (*n*) for each experimental group/condition, given as a discrete number and unit of measurement
☐	☒	A statement on whether measurements were taken from distinct samples or whether the same sample was measured repeatedly
☐	☒	The statistical test(s) used AND whether they are one- or two-sided<br>*Only common tests should be described solely by name; describe more complex techniques in the Methods section.*
☒	☐	A description of all covariates tested
☒	☐	A description of any assumptions or corrections, such as tests of normality and adjustment for multiple comparisons
☒	☐	A full description of the statistical parameters including central tendency (e.g. means) or other basic estimates (e.g. regression coefficient) AND variation (e.g. standard deviation) or associated estimates of uncertainty (e.g. confidence intervals)
☐	☒	For null hypothesis testing, the test statistic (e.g. *F*, *t*, *r*) with confidence intervals, effect sizes, degrees of freedom and *P* value noted<br>*Give P values as exact values whenever suitable.*
☐	☒	For Bayesian analysis, information on the choice of priors and Markov chain Monte Carlo settings
☒	☐	For hierarchical and complex designs, identification of the appropriate level for tests and full reporting of outcomes
☒	☐	Estimates of effect sizes (e.g. Cohen's *d*, Pearson's *r*), indicating how they were calculated

Our web collection on statistics for biologists contains articles on many of the points above.

Software and code

Policy information about availability of computer code

Data collection	For identification of the Q586B2 the available trypanosome genome (Trypanosoma brucei brucei TREU 927) within the Well-come Trust Sanger Institute database (http://www.sanger.ac.uk/resources/databases) was used. The Protein Homology/analogY Recognition Engine Version 2.0 (Phyre2) (http://www.sbg.bio.ic.ac.uk/phyre2/html/page.cgi?id=index), was used for analyzing structural homologies between proteins.<br>The NCBI and TriTrypDB databases were used for screening a wide range of eukaryote genomes. Protein purification was performed using an Akta Explorer 10S (GE Healthcare) system. ELISA data were collected via an ELX808 Absorbance Microplate Reader (BioTek Instruments, Winooski, VT, USA).

Data analysis	ColabFold predictions (https://www.alphafold.ebi.ac.uk) was used to predict the structure of Q586B2 and for comparison with the known structure of monomeric Q4D6Q6. The prediction code used for the prediction was that of Q4D6Q615 with the crystal structure (PDB code 6xyb).<br>The GraphPad Prism 7 software was used for statistical analyses of the data. FlowJo software 10 (Tree Star Inc., Ashland, OR) was used for analyzing FACS data. Quantification of the Wester blot was performed using a Vilber Fusion Imager (Viber) and Fusion FX6 Edge 18.05 software. The fluorescent images were analysed using the Zeiss Axio Imager M2 wide field software. For the phylogenetic tree the following data anlysis tools were used:<br>- MAFFT v7 (Katoh et al. 2019): sequence alignment<br>- MrBayes 3.2.7a (Ronquist et al. 2012) as implemented in the CIPRES science gateway 3.3 (Miller et al. 2010): Bayesian phylogeny inference<br>- Tracer 1.5 (Rambaut & Drummond, 2009): to verify if the MrBayes analyses had reached effective sampling sizes > 200 for all model parameters. ELISA data were obtained via an ELX808 Absorbance Microplate Reader (BioTek Instruments, Winooski, VT, USA) and Gen5 1.08 software (BioTek Instruments). Chromatograms of protein purification (cf. Akta Explorer10s system) were obtained via Unicorn 5.1 software.

For manuscripts utilizing custom algorithms or software that are central to the research but not yet described in published literature, software must be made available to editors and reviewers. We strongly encourage code deposition in a community repository (e.g. GitHub). See the Nature Portfolio guidelines for submitting code & software for further information.

Data

Policy information about availability of data

All manuscripts must include a data availability statement. This statement should provide the following information, where applicable:

- Accession codes, unique identifiers, or web links for publicly available datasets
- A description of any restrictions on data availability
- For clinical datasets or third party data, please ensure that the statement adheres to our policy

Source data are provided within this paper. Genes used for the phylogenetic tree (Fig. S1 and S2) are labelled by their accession number in TRiTrypDB (numbers without asterisk) or NCBI Genbank (numbers with asterisk) and are shown in Table S1. Nanobody sequences have been provided within Fig. S4a and part of the data presented in this work can be accessed in the patent application PCT/EP2022/058575.

Research involving human participants, their data, or biological material

Policy information about studies with human participants or human data. See also policy information about sex, gender (identity/presentation), and sexual orientation and race, ethnicity and racism.

Reporting on sex and gender	No human samples of subjects were used in this study
Reporting on race, ethnicity, or other socially relevant groupings	No human samples of subjects were used in this study
Population characteristics	No human samples of subjects were used in this study
Recruitment	No human samples of subjects were used in this study
Ethics oversight	No human samples of subjects were used in this study

Note that full information on the approval of the study protocol must also be provided in the manuscript.

Field-specific reporting

Please select the one below that is the best fit for your research. If you are not sure, read the appropriate sections before making your selection.

☒ Life sciences ☐ Behavioural & social sciences ☐ Ecological, evolutionary & environmental sciences

For a reference copy of the document with all sections, see nature.com/documents/nr-reporting-summary-flat.pdf

Life sciences study design

All studies must disclose on these points even when the disclosure is negative.

Sample size	The sample size of every individual group within every single experiment is indicated in the individual figure legends. For flow cytometry and ELISA as well as immunofluorescence microscopy experiments, a sample size of at least 3 per experimental condition were conducted. Sample sizes were determined based on the feasibility of obtaining sufficient biological replicates to ensure robustness and statistical significance of our findings. While no formal sample size calculation was conducted due to the explorative nature of this study we adopted a sample size that aligns with common practice in similar experiments conducted in the lab. Specifically, the sample sizes were chosen to ensure a representative capture of biological variability and the ability to identify significant differences in Nanobody signals or biological responses across the experimental conditions.
Data exclusions	No data were excluded from the samples

Replication	All experiments were performed at least 2-3 times.
Randomization	Randomization was not performed due to the nature of the study, which involved time course analysis of infection progression. Instead, efforts were made to control potential confounding factors by ensuring uniformity in the age, sex and genetic background of the mice used in each of the groups. For the infection of mice with different parasite strains we ensured that within each experiment mice of the same age/sex and gender was used. The same approach was used for the Nanobody treatment experiments.
Blinding	Blinding was not performed in this study. Yet, rigorous quality control measures and standardized procedures were employed to ensure the reliability and reproducibility of the results.

Behavioural & social sciences study design

All studies must disclose on these points even when the disclosure is negative.

Study description	N/A
Research sample	N/A
Sampling strategy	N/A
Data collection	N/A
Timing	N/A
Data exclusions	N/A
Non-participation	N/A
Randomization	N/A

Ecological, evolutionary & environmental sciences study design

All studies must disclose on these points even when the disclosure is negative.

Study description	N/A
Research sample	N/A
Sampling strategy	N/A
Data collection	N/A
Timing and spatial scale	N/A
Data exclusions	N/A
Reproducibility	N/A
Randomization	N/A
Blinding	N/A

Did the study involve field work? ☐ Yes ☒ No

Field work, collection and transport

Field conditions	N/A
Location	N/A
Access & import/export	N/A
Disturbance	N/A

Reporting for specific materials, systems and methods

We require information from authors about some types of materials, experimental systems and methods used in many studies. Here, indicate whether each material, system or method listed is relevant to your study. If you are not sure if a list item applies to your research, read the appropriate section before selecting a response.

Materials & experimental systems

n/a	Involved in the study
☐	☒ Antibodies
☒	☐ Eukaryotic cell lines
☒	☐ Palaeontology and archaeology
☐	☒ Animals and other organisms
☒	☐ Clinical data
☒	☐ Dual use research of concern
☒	☐ Plants

Methods

n/a	Involved in the study
☒	☐ ChIP-seq
☐	☒ Flow cytometry
☒	☐ MRI-based neuroimaging

Antibodies

Antibodies used

The following anti-mouse antibodies were used:
- For flow cytometry:
The anti-VSG Nb (Nb33) and all anti-Q586B2 Nbs were generated in-house, Rat anti-mouse CD16/CD32 (Mouse Fc Block, Clone 2.4G2 kind gift from Louis Boon (JJP Biologicals)), Alexa Fluor ® 488 labelled anti-HA IgG (BioLegend, clone 16B12, A488-101L), APC/Cyanine7 anti-mouse CD45 (BioLegend, clone 30-F11, 103116), PE/Cyanine7 anti-mouse/human CD11b (BioLegend, clone M1/70, 101216), Brilliant Violet 510™ anti-mouse/human CD11b (BioLegend, clone M1/70, 101263), APC anti-mouse Ly-6C (BioLegend, clone AL-21, 560595), PerCP/Cyanine5.5 anti-mouse Ly-6G (BioLegend, clone 1A8, 127616), PE-Cy™7 Rat Anti-Mouse Ly-6G (BD Bioscience, clone 1A8, 560601), PE anti-mouse F4/80 (BioLegend, clone BM8, 123110), FITC anti-mouse F4/80 (BioRad, clone Cl:A3-1, MCA497FB), Brilliant Violet 421™ anti-mouse I-A/I-E (BioLegend, clone M5/114.15.2, 107632), Brilliant Violet 510™ anti-mouse CD8a (BioLegend, clone 53-6.7, 100752), PE-Cyanine7 anti-mouse CD90.1(Thermo Fisher Scientific, clone Thy-1.1 (HIS51), 14-0900-82), Brilliant Violet 510™ anti-mouse CD19 (BioLegend, clone 6D5, 115545), FITC anti-mouse CD4 (BioLegend, clone RM4-5, 100509), PE rat anti-mouse NK1.1 (Thermo Fisher Scientific, clone PK136, 12-5941-82), PE rat anti-mouse SiglecF (BD Bioscience, clone E50-2440, 552126), 7-AAD BD (BD Pharmingen, clone RUO, 559925).
- Fluorescent microscopy:
Nb39 was used at 5 µg/stain. The goat anti-V5 IgG (clone, R960-25, 1:100 dilution), mouse anti-p67 IgG (1:1000 dilution), rabbit anti-RAB2B (1:500), rabbit anti-RAB5B (1:500), rabbit anti-RAB11(1:500), anti-VIT1 (1:500), anti-GK (1:1000) and rabbit anti-BiP (1:1000 dilution) were provided by Dr. James Bangs (University of Buffalo). For detection, a Donkey anti-Goat IgG (H+L) IgG Cross-Adsorbed Secondary Antibody PE (Thermo Fisher Scientific, PA1-29953), Goat anti-Mouse IgG1 Cross-Adsorbed Secondary Antibody PE (Thermo Fisher Scientific, P-21129) or Goat anti-Rabbit IgG (H+L) Cross-Adsorbed Secondary Antibody PE (Thermo Fisher Scientific, P-2771MP) were used at 1/1000 dilution. Alexa Fluor ® 488 labelled anti-HA IgG (BioLegend, clone 16B12, A488-101L), DAPI (Sigma Aldrich, 1/1000, 28718-90-3).
-Western blotting:
Primary antibodies for PAD1 and EF1α (kindly gifted by Prof. Keith Matthews, University of Oxford) were used at 1:1000 and 1:7000, respectively. For detection, an HRP-coupled goat anti-Rabbit IgG (H+L) (1/1000, Thermo Fisher Scientific) or goat anti-Mouse IgG (H +L) (1/1000, NovusBio) were used.

Validation

All antibodies used in this study were validated for species specificity and application suitability. Validation information, including specificity and recommended applications, was obtained from the manufacturer's websites and technical documentation. Concentrations were decided according to the recommendations of the manufacturer and tested in pilot titration experiments.

Eukaryotic cell lines

Policy information about cell lines and Sex and Gender in Research

Cell line source(s)	N/A
Authentication	N/A
Mycoplasma contamination	N/A
Commonly misidentified lines (See ICLAC register)	N/A

Palaeontology and Archaeology

Specimen provenance	N/A

Specimen deposition	N/A
Dating methods	N/A

☐ Tick this box to confirm that the raw and calibrated dates are available in the paper or in Supplementary Information.

Ethics oversight	N/A

Note that full information on the approval of the study protocol must also be provided in the manuscript.

Animals and other research organisms

Policy information about studies involving animals; ARRIVE guidelines recommended for reporting animal research, and Sex and Gender in Research

Laboratory animals	Eight weeks old female C57BL/6 mice were purchased from Janvier, France. Vert-X (B6(Cg)-Il10tm1.1Karp/J) were purchased from Jackson Laboratory, USA. LysMCre * IL-10fl/fl (i.e. LysM-IL10) mice were generated in house by crossing the LysMCre (B6.129P2-Lyz2tm1(cre)Ifo/J, JAX stock #004781) mice with IL-10fl/fl mice (a kind gift of W. Muller, University of Manchester, Manchester, United Kingdom). For T. cruzi infections, BALB/cJ male mice (60-day-old obtained from Jackson laboratories). Animals were housed in IVCs or open cages with appropriate cage enrichment and fed ad libitum. A 12-hour light/dark cycle was maintained, with ambient temperature at 20-24°C and humidity levels between 40-60%.
Wild animals	No wild animals were used in this study.
Reporting on sex	Mainly female mice were used in this study, except for experiments using T. cruzi (performed in S. America). Female mice are often used in research because they are less aggressive than males, reducing the likelihood of injuries that could affect experimental results. Their social structure (they can be housed in groups without fighting) also makes it easier to manage them in a laboratory setting. Additionally, the estrous cycle in female mice is much shorter and more regular than the menstrual cycle, offering more consistency in<br>experimental conditions. Reason for using male mice when performing T. cruzi infections is that this gender exhibits a stronger response towards the parasite causing early mortality. In this study the most aggressive model was used to validate the vaccination potential.
Field-collected samples	No field samples were used in this study.
Ethics oversight	All experiments complied with the ECPVA guidelines (CETS n° 123) and were approved by the VUB Ethical Committee (Permit Number: 17-220-02). The protocol of animal procedures followed in this study was approved by the Ethics Committee of Animal Experiments of the Universidad Nacional de San Martín (CICUAE N° 14/2022). Mice were monitored daily. Humane endpoints were used during the study, based on weight loss, animals with >25% weight loss were sacrificed using carbon dioxide treatment.

Note that full information on the approval of the study protocol must also be provided in the manuscript.

Clinical data

Policy information about clinical studies

All manuscripts should comply with the ICMJEguidelines for publication of clinical research and a completedCONSORT checklist must be included with all submissions.

Clinical trial registration	N/A
Study protocol	N/A
Data collection	N/A
Outcomes	N/A

Dual use research of concern

Policy information about dual use research of concern

Hazards

Could the accidental, deliberate or reckless misuse of agents or technologies generated in the work, or the application of information presented in the manuscript, pose a threat to:

No	Yes	
[x]	[ ]	Public health
[x]	[ ]	National security
[x]	[ ]	Crops and/or livestock
[x]	[ ]	Ecosystems
[x]	[ ]	Any other significant area

Experiments of concern

Does the work involve any of these experiments of concern:

No	Yes	
[x]	[ ]	Demonstrate how to render a vaccine ineffective
[x]	[ ]	Confer resistance to therapeutically useful antibiotics or antiviral agents
[x]	[ ]	Enhance the virulence of a pathogen or render a nonpathogen virulent
[x]	[ ]	Increase transmissibility of a pathogen
[x]	[ ]	Alter the host range of a pathogen
[x]	[ ]	Enable evasion of diagnostic/detection modalities
[x]	[ ]	Enable the weaponization of a biological agent or toxin
[x]	[ ]	Any other potentially harmful combination of experiments and agents

Plants

Seed stocks	N/A
Novel plant genotypes	N/A
Authentication	N/A

ChIP-seq

Data deposition

[ ] Confirm that both raw and final processed data have been deposited in a public database such as GEO.

[ ] Confirm that you have deposited or provided access to graph files (e.g. BED files) for the called peaks.

Data access links<br>*May remain private before publication.*	N/A
Files in database submission	N/A
Genome browser session<br>(e.g. UCSC)	N/A

Methodology

Replicates	N/A
Sequencing depth	N/A
Antibodies	N/A
Peak calling parameters	N/A
Data quality	N/A
Software	N/A

Flow Cytometry

Plots

Confirm that:

☒ The axis labels state the marker and fluorochrome used (e.g. CD4-FITC).

☒ The axis scales are clearly visible. Include numbers along axes only for bottom left plot of group (a 'group' is an analysis of identical markers).

☒ All plots are contour plots with outliers or pseudocolor plots.

☒ A numerical value for number of cells or percentage (with statistics) is provided.

Methodology

Sample preparation	*Describe the sample preparation, detailing the biological source of the cells and any tissue processing steps used.*
Instrument	*Identify the instrument used for data collection, specifying make and model number.*
Software	*Describe the software used to collect and analyze the flow cytometry data. For custom code that has been deposited into a community repository, provide accession details.*
Cell population abundance	*Describe the abundance of the relevant cell populations within post-sort fractions, providing details on the purity of the samples and how it was determined.*
Gating strategy	*Describe the gating strategy used for all relevant experiments, specifying the preliminary FSC/SSC gates of the starting cell population, indicating where boundaries between "positive" and "negative" staining cell populations are defined.*

☒ Tick this box to confirm that a figure exemplifying the gating strategy is provided in the Supplementary Information.

Magnetic resonance imaging

Experimental design

Design type	N/A
Design specifications	N/A
Behavioral performance measures	N/A

Acquisition

Imaging type(s)	N/A
Field strength	N/A
Sequence & imaging parameters	N/A
Area of acquisition	N/A

Diffusion MRI ☐ Used ☒ Not used

Preprocessing

Preprocessing software	N/A
Normalization	N/A
Normalization template	N/A
Noise and artifact removal	N/A
Volume censoring	N/A

Statistical modeling & inference

Model type and settings	N/A

Effect(s) tested | N/A

Specify type of analysis: ☐ Whole brain ☐ ROI-based ☐ Both

Statistic type for inference (See Eklund et al. 2016) | N/A

Correction | N/A

Models & analysis

n/a	Involved in the study
☒	☐ Functional and/or effective connectivity
☒	☐ Graph analysis
☒	☐ Multivariate modeling or predictive analysis

Functional and/or effective connectivity | N/A

Graph analysis | N/A

Multivariate modeling and predictive analysis | N/A
